# Functional Suitability Evaluation of Radioimmunoconjugates of Nanobodies against PD-L1 and HER2/neu for Tumor Theranostics

**DOI:** 10.17691/stm2026.18.2.03

**Published:** 2026-04-30

**Authors:** K.O. Avrov, S.V. Shatik, O.A. Shashkova, A.A. Pinevich, L.A. Terekhina, I.V. Gryazeva, V.V. Zaitsev, M.A. Berlina, A.A. Stanzhevsky, M.P. Samoylovich

**Affiliations:** PhD, Senior Researcher, Laboratory of Hybridoma Technology; Russian Scientific Center of Radiology and Surgical Technologies named after Academician A.M. Granov, Ministry of Health of the Russian Federation, 70 Leningradskaya St., Saint Petersburg, Pesochny Settlement, 197758, Russia; PhD, Leading Researcher, Laboratory of Radiopharmaceutical Technologies; Russian Scientific Center of Radiology and Surgical Technologies named after Academician A.M. Granov, Ministry of Health of the Russian Federation, 70 Leningradskaya St., Saint Petersburg, Pesochny Settlement, 197758, Russia; PhD, Senior Researcher, Laboratory of Hybridoma Technology; Russian Scientific Center of Radiology and Surgical Technologies named after Academician A.M. Granov, Ministry of Health of the Russian Federation, 70 Leningradskaya St., Saint Petersburg, Pesochny Settlement, 197758, Russia; PhD, Senior Researcher, Laboratory of Hybridoma Technology; Russian Scientific Center of Radiology and Surgical Technologies named after Academician A.M. Granov, Ministry of Health of the Russian Federation, 70 Leningradskaya St., Saint Petersburg, Pesochny Settlement, 197758, Russia; Researcher, Laboratory of Hybridoma Technology; Russian Scientific Center of Radiology and Surgical Technologies named after Academician A.M. Granov, Ministry of Health of the Russian Federation, 70 Leningradskaya St., Saint Petersburg, Pesochny Settlement, 197758, Russia; PhD, Senior Researcher, Laboratory of Hybridoma Technology; Russian Scientific Center of Radiology and Surgical Technologies named after Academician A.M. Granov, Ministry of Health of the Russian Federation, 70 Leningradskaya St., Saint Petersburg, Pesochny Settlement, 197758, Russia; Leading Researcher, Laboratory of Radiopharmaceutical Technologies; Russian Scientific Center of Radiology and Surgical Technologies named after Academician A.M. Granov, Ministry of Health of the Russian Federation, 70 Leningradskaya St., Saint Petersburg, Pesochny Settlement, 197758, Russia; Research Assistant, Laboratory of Hybridoma Technology; Russian Scientific Center of Radiology and Surgical Technologies named after Academician A.M. Granov, Ministry of Health of the Russian Federation, 70 Leningradskaya St., Saint Petersburg, Pesochny Settlement, 197758, Russia; MD, DSc, Deputy Director for Research; Russian Scientific Center of Radiology and Surgical Technologies named after Academician A.M. Granov, Ministry of Health of the Russian Federation, 70 Leningradskaya St., Saint Petersburg, Pesochny Settlement, 197758, Russia; DSc, Chief Researcher, Laboratory of Hybridoma Technology; Russian Scientific Center of Radiology and Surgical Technologies named after Academician A.M. Granov, Ministry of Health of the Russian Federation, 70 Leningradskaya St., Saint Petersburg, Pesochny Settlement, 197758, Russia; Chief Researcher, Department of Cytology and Histology, Faculty of Biology; Saint Petersburg State University, Universitetskaya Embankment, 7/9 Saint Petersburg, 199034, Russia

**Keywords:** radioimmunoconjugates, nanobodies, radionuclides, PD-L1, HER2/neu, theranostics

## Abstract

**The aim of the study** was to evaluate the applicability of radioimmunoconjugates (RIC) of nanobodies against PD-L1 and HER2/ neu for the diagnosis and therapy of malignant tumors.

**Materials and Methods:**

The nanobodies to human biomarkers PD-L1 and HER2/neu were conjugated with radionuclides ^68^Ga and ^177^Lu using the chelating agent DOTA. RIC biodistribution was studied in experimental models on F1 (DBA/2xBALB/c) mice, which were inoculated with genetically modified CT26 murine carcinoma cells expressing human PD-L1 or HER2/neu. RICs containing the isotope ^68^Ga and intended for tumor detection were administered to animals intravenously at a dose of 1.0–1.2 MBq. In 0.5, 1.5, and 4 h the radioactivity accumulation in specific tumors carrying human biomarkers was assessed using direct dosimetry compared with control tumors. RICs containing ^177^Lu were administered for therapy at a dose of 0.8–1.6 MBq, and studied similarly for 96 h after administration. **Results.** After RICs containing ^68^Ga were administered to mice, the greatest difference between specific and control tumors was observed in 1.5 h. At the same time, there was a multiple excess of the specific tumor radioactivity over the blood and muscle tissue radioactivity, which should provide high contrast imaging. After administering RICs containing ^177^Lu to mice, the radioactivity in specific tumors persisted for 48 h producing a long-term effect of the radioisotope on the tumor. With the introduction of all RICs, there was a rapid elimination of radioactivity from the blood through urine, which was associated with the use of nanobodies with a molecular weight of 13 kDa and not containing sites of interaction with Fc receptors. There were found the significant accumulation and long-term retention of radioactivity in the kidneys. The work revealed a dependence of the radioactivity biodistribution on the isotope used: the time-normalized radioactivity of some organs and tissues, including tumors, after the administration of RICs containing ^68^Ga appeared to be higher than after administering the RICs of the same specificity, but containing ^177^Lu radioisotopes.

**Conclusion:**

The findings indicated the functional suitability of the pairs of RIC nanobodies with ^68^Ga and ^177^Lu radioisotopes for theranostics of malignant tumors expressing PD-L1 and HER2/neu biomarkers.

## Introduction

The use of selective radioimmunoconjugates specific to biomarkers has opened up new possibilities for cancer diagnostics and therapy. A radionuclide in such radiopharmaceuticals is attached to a target directed vector: the whole monoclonal antibody, its fragment, a scaffold protein, a peptide ligand, or any small molecule. In radionuclide therapy, theranostics is the use of a pair of radiopharmaceuticals bind to one molecular target; one of the pharmaceutical pair is used for tumor diagnostics, while another — for tumor therapy. Theranostics opens great opportunities for personalized tumor therapy enabling well in advance to assess the efficiency of a certain approach in patient’s treatment [1]. In the past decade, great progress has been achieved in tumor theranostics. It is primarily associated with using somatostatin receptor (SSTR) ligands, as well as prostate-specific membrane antigen (PSMA) ligands labeled with radionuclides ^68^Ga and ^177^Lu [2–4].

PD-L1 and HER2/neu proteins are well-studied tumor biomarkers. PD-L1 is the ligand of programmed cell death receptor PD1. It is constitutively expressed in a number of immune cells, in some epithelial cells, and in many tumor cells. The protein is one of the key control points of immunity used by tumor cells to evade an immune response. PD-L1 binding on the surface of antigen-presenting cells to receptor PD1 expressed by T lymphocytes results in T lymphocyte proliferation and cytotoxicity suppression and their production of cytokines [5, 6]. Furthermore, PD-L1 expression on the tumor cell surface can block an immune response promoting tumor growth [7–9]. HER2/neu (type 2 human epidermal growth factor receptor) tyrosine kinase is a protein belonging to the epidermal growth factor receptor family. The protein heterodimerization with other family members, as well as its homodimerization, result in the activation of signal cascades and cell proliferation [10–12]. In breast cancer, gastric cancer, ovarian carcinoma, lung cancer, and uterine cancer, frequently, there is high expression of HER2/neu leading to its dimerization, and, as a consequence, the sustained activation of signal pathways [10]. The tumors with PD-L1 and HER2/ neu hyperexpression are treated with blocking these biomarkers by monoclonal antibodies, and antibody-drug conjugates are also used [8, 13].

PD-L1 and HER2/neu hyperexpression are associated with aggressive development of the disease; moreover, it is possible to use the conjugates specifically binding to these biomarkers for tumor therapy. No marked expression of PD-L1 and HER2/neu on tumor cell surface makes the selective action on the proteins inappropriate. Thus, determining PD-L1 and HER2/ neu expression levels is of great prognostic value and necessary in choosing therapy. Invasive biopsy followed by immunohistochemistry is used to assess an expression level, it being a standard practice in patients’ treatment. However, the method fails to consider spatial heterogeneity and time dynamics of PD-L1 and HER2/neu expression. Therefore, the non-invasive determination of the status of the biomarkers using imaging techniques, primarily positron-emission tomography, is the significant supplement for research using immunohistochemistry.

Creating theranostic radiopharmaceutical pairs for diagnostics and therapy of the tumors specifically binding to PD-L1 or HER2/neu is a crucial task. The present study investigated radioimmunoconjugates (RIC) based on the antibodies against PD-L1 and HER2/neu. The nanobodies weighing 12–15 kDa are antigen binding VHH fragments formed only by heavy chains of IgG antibodies found in paridigitate mammals, *Camelidae*. These nanobodies are also called VHH antibodies. The nanobodies weighing 12–15 kDa exhibit less immunogenicity and significantly greater stability than whole monoclonal antibodies weighing 150 kDa [14]. Since nanobodies are not glycated, they can be obtained, unlike monoclonal antibodies, in prokaryotes, and in that way decreasing production costs. CDR3 domain loop in nanobodies is longer than in standard IgG that along with a small size enables nanobodies to bind to hidden epitopes [15]. In recent years there have been published the works on studying the radiopharmaceuticals based on nanobodies, including the reports on RIC antibodies against PD-L1 and HER2/neu [16–23].

When using a theranostic pair of radiopharma-ceuticals, it is desirable they would differ in radionuclides only, their vector molecules being the same. The approach helps the standardization and simplifies the logistics of radioconjugate production that improves therapy quality and reduces its cost. We synthesized two theranostic RIC pairs, each pair being based on one nanobody: ^68^Ga-VHH PD-L1 and ^177^Lu-VHH PD-L1, ^68^Ga-VHH HER2/neu and ^177^Lu-VHH HER2/neu. In their production we used one chelating agent DOТA and radionuclides ^68^Ga and ^177^Lu. Gallium-68 is an isotope, its half-life being 68 min, radiating positrons and used for diagnostics by positron emission tomography [24]. Lutetium-177 is an isotope, its half-life being 6.65 days; it is the source of β-radiation and used for tumor therapy [25].

**The aim of the study** was to investigate the functional suitability (applicability) of RIC of nanobodies against PD-L1 and HER2/neu for the diagnosis and therapy of malignant tumors. For this purpose, we determined the specificity of RIC binding to a target tumor antigen and RIC biodistribution dynamics in mice.

## Materials and Methods

### Human PD-L1 and HER2/neu affinity antibodies

The research objects were conjugated with radioisotopes, recombinant single-domain heavy-chain antibodies (nanobodies, VHH) recognizing human epitope PD-L1 (VHH-PD-L1) and HER2/neu (VHH-HER2/neu) (provided by Innova plus, Russia).

### RIC synthesis

To obtain chelating precursors, we used specific to PD-L1 and HER2/neu biomarkers and commercially available bifunctional chelating agent p-SCN-Bn_DOTA (Macrocyclics, USA). To synthesize RIC containing ^68^Ga, we used eluate of gallium-68 generator (Cyclotron, Russia) in the form of gallium chloride solution in hydrochloric acid, 0.1 M. 100 μl of 50 mM ammonium-acetate buffer (pН 7.5) and 100 μl of the generator eluate containing 30–50 MBq ^68^Ga were added to the chelating precursor (100 μg) dissolved in 50 mM ammonium-acetate buffer, 20 μl (pН 7.5). The reaction mixture was incubated for 15 min using the thermal shaker TS-100C (SIA Biosan, Latvia) at 1000 rpm, at 70°C in a protective laminar box. The specific activity of RIC was 0.3–0.5 MBq/μg.

To obtain RIC containing ^177^Lu we used lutecium chloride solution (^177^Lu) without a carrier in 0.1 М HCl (RIAR JSC ROSATOM, Russia). The chelating precursor, 100 μg, was dissolved by 50 mM ammonium acetate solution, 100 μl. The resulting mixture was acidified with 22.5 μl of 0.1 M hydrochloric acid up to the pH ~5.5–5.8, and [^177^Lu]LuCl_3_ (15–45 MBq, 2.5 μl) was added. ^177^Lu was radiolabeled in a closed tube at 37°C within 60 min in the presence of atmospheric oxygen, stirred in a thermal shaker (1000 rpm) in a protective laminar box. The obtained RICs were studied using radio-thin-layer chromatography and ITLC SG plates (Agilent, USA) and radio-high performance liquid chromatography on the chromatographer Ultimate 3000 (Dionex, USA) and the column Superdex 75 Increase (Cytiva, USA). The specific activity of RIC was 0.15–0.45 MBq/μg. The radiochemical purity of all RICs was not less than 95%.

### Assessment of specific RIC binding to antigens using magnetic particles

Specific activity of RICs was determined as described above [26] using commercially available magnetic protein A-covered particles (SureBeads, No.161-4013, Bio-Rad, USA; MagBeads, No.L00273, MagBio Genomics, USA; or SileksMag, No.K0181, SileksMag, Russia). For this purpose, the specific antigen conjugated with Fc fragment (PD-L1-Fc or HER2/neu-Fc) was immobilized on magnetic particles, followed by the particles being incubated with RIC solution (0.5–4.0 μkg/ml). After washing the particles free from unbound RIC, their radioactivity was measured using a radiation meter of radionuclide activity Triathler and the software Triathler Becquerel Finder (Hidex, Finland). For control, RICs were incubated with the particles, which were not covered by an antigen. The specific RIC binding parameter was the radioactivity portion bound to antigen-covered particles from the radioactivity initially added to the incubation test specimen. *In vivo* testing was for those RICs, in which the radioactivity portion specifically bound to target proteins on magnetic particles was 70–90% from the radioactivity added to the specimen.

### Biological models for *in vivo* RIC testing

RIC biodistribution dynamics was studied using the biological models we developed. VHH-PD-L1-based RICs were tested using CT26-PD-L1 strain cells, which were genetically modified BALB/c mice CT26 colon cancer cells expressing the human PD-L1 membrane. The development and characteristics of a biological model based on these cells were previously described [27]. To assess the biodistribution dynamics of RICs based on VHH-HER2/neu antibodies, we used CT26-HER2/ neu strain cells, which were also obtained by gene-engineering technique. Their production and main characteristics are given down below.

### A biological model for testing VHH-HER2/neu-based RIC

**Figure 1.**
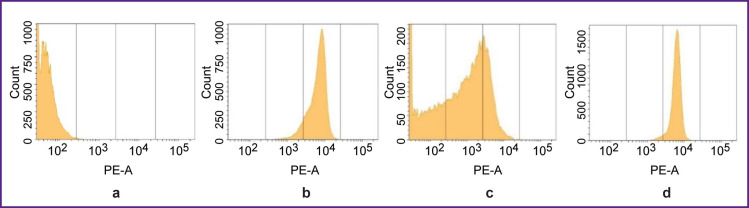
Revealing human HER2/neu expression by flow immunocytofluorometry on parent murine CT26 cells (a) and on recombinant CT26-HER2/neu cells in culture (b); in the cell suspension of the tumor taken out on day 15 after CT26-HER2/neu cells were transplanted to the mice (c), and the same cell suspension from the tumor after 11-day *in vitro* culture (d)

The CT26-HER2/neu strain was obtained by retroviral transduction of CT26 murine carcinoma cells with the pQCXIP-HER2/neu plasmid. Human melanoma MeWo cells served as the *HER2/neu* cDNA donor to create a vector. CT26-HER2/neu strain cells, unlike murine parent CT26 cells, exhibited the high expression of human HER2/neu gene and protein that was proved by the polymerase chain reaction in a real-time mode and flow immunocytofluorometry (Figure 1). The strain had high stability and maintained the initial biomarker expression level when in culture within 15 passages (a follow-up period) — both in the presence of a selective antibiotic, and in its absence. CT26-HER2/ neu cells (5 million) subcutaneously administered to the sublethally irradiated mice F1(DBA/2xBALB/c) on days 9–11 resulted in forming solid tumors, 100–500 mm3 in volume, in the animals. The content of cells, which had human HER2/neu on their membrane, on day 15–17 of tumor growth, was 30–40% (Figure 1 (b)). After the explantation and *in vitro* culture of cells from these tumors within 11 days, the content of HER2/neu-positive cells increased up to 99% indicating the high stability of the biomarker expression by CT26-HER2/neu strain cells (Figure 1 (c)). The number of HER2/neu molecules determined by binding with RIC was 1.6 million per cell.

### Experiments on animals

The experiments were carried out on F1(DBA/2×BALB/c) mice of both genders. The animals were kept in standard conditions in accordance with European Parliament and European Union Council Direction on protection of animals used for scientific purposes 2010/63/EU dated September 22, 2010. The study was approved by the Ethics Committee of Russian Scientific Center of Radiology and Surgical Technologies named after Academician A.M. Granov (Saint Petersburg, Russia). On the day the experiment started the mice were exposed to therapeutic radiation (dose: 5 Gy). The dose was decided upon in a series of trial experiments aimed at reducing the immunoresponsiveness of the animals in relation to human antigens. The mice exposed to 5 Gy-radiation resulted in no animal death within 6 months. In control experiments non-irradiated intact mice were used. CТ26-PD-L1 or CT26-HER2/neu cells stably expressing human biomarkers on the membrane were grafted subcutaneously in the right side: 5·106 cells per mouse. A million control CT26 cells, which expressed no human biomarkers, were injected subcutaneously into the left side.

### RIC biodistribution dynamics study by direct radiometry

The study involved the mice on days 9–13 after transplanting the tumor cells. The animals were anesthetized (30 mg/kg Zoletil 100 and 8 mg/ kg XylaVET intramuscularly) followed by injecting RIC solutions (100 μl) into the retro-orbital venous sinus. The volume activity of RICs containing ^68^Ga was 10–12 MBq/ ml, ^177^Lu — 8–16 MBq/ml.

The biodistribution was studied in the experimental animals 0.5, 1.5, 4, 24, 48, and 96 h after administering the antigens conjugated with a radioisotope ^177^Lu (the half-life period is 6.65 days), or 0.5, 1.5, and 4 h after ^68^Ga-labeled antibodies (half-life period is 68 min) administered. The mice were sacrificed by injecting an overdose of Zoletil 100 followed by the autopsy, the tissue and biological fluid samples taken. The activity of radionuclides in the samples was quantitatively determined by a radioactivity meter Triathler (Hidex, Finland) using the energy window with 150–350 channels — for ^68^Ga, and 0–150 channels — for ^177^Lu. The radioactivity values of the recuperated organs and tissues were recounted for the autopsy time (zero time point) according to the formula: *A*_0_=*AТ*_1_·е^(Ln(2)·(*T*_1_–*T*_0_)/*T*_1/2_), where *A*_0_ — the sample activity at autopsy time (Bq); *AТ*_1_ — the sample activity when measuring the activity (Bq); *T*_1_ — activity measuring time (h); *T*_0_ — autopsy time (h); *T*_1/2_ — half-time period (h). Then the tissue radioactivity was calculated in percentage of the administered activity calculated with reference to 1 g of tissue.

### Statistical processing of data

For statistical processing of the experimental findings, we applied GraphPad Prizm 10. The findings were represented in the form of bar graphs with the indication of a standard deviation for each mean value (m±σ). Mann–Whitney U-test was used to assess the differences between the two independent samples. The results were considered statistically significant in p≤0.05. All results were obtained in no less than three repeats.

## Results

**Figure 2.**
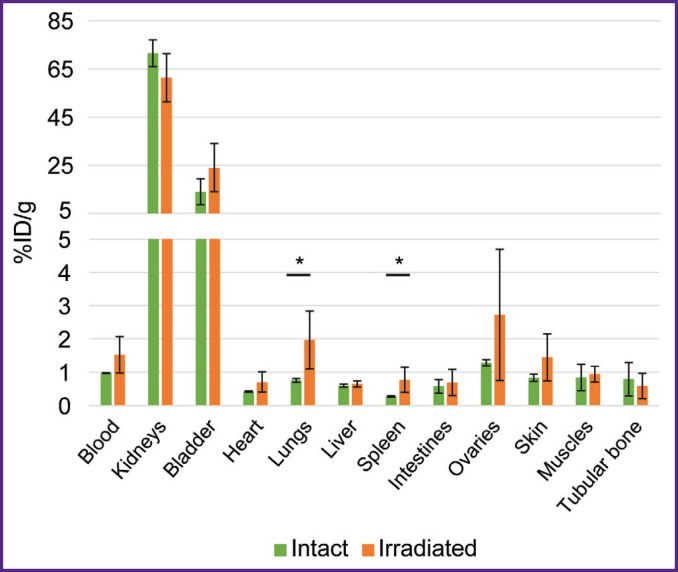
Biodistribution of radioimmunoconjugates ^68^Ga-VHH HER2/neu 1 h after being administered to the intact mice and the mice irradiated (dose 5 Gy) 8 days before autopsy Along Y-axis — radioactivity of 1 g tissue, the portion in percentage of the administered radioactivity. The results are represented as m±σ; * p<0.05

To study RIC biodistribution, we developed human tumor models. For this purpose, F1(DBA/2xBALB/c) mice were administered genetically modified CT26-PD-L1 or CT26-HER/neu cells expressing the human tumor biomarkers PD-L1 or HER2/neu. The same animals were inoculated with a control tumor by administering murine CТ26 cells. To form tumors, the mice immunoreactivity was preliminarily reduced by a sublethal X-ray dose (5 Gy). There was found no X-ray radiation effect on RIC biodistribution: after administering ^68^Ga-VHH HER2/neu, the radioactivity accumulation in the organs and tissues of the mice irradiated 8 days prior to the autopsy did not differ from the accumulation in those non-irradiated (Figure 2). Similar findings were obtained after administering ^177^Lu-VHH PD-L1 (the data not presented).

**Figure 3.**
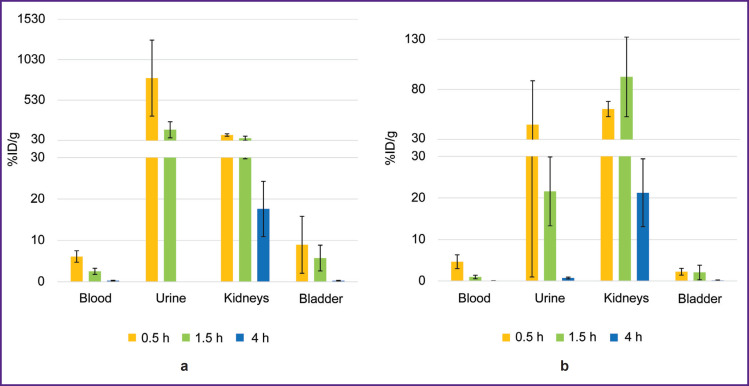
Change in the radioactivity of blood, urine, the kidneys, and the urinary bladder after administering radioimmunoconjugates: (a) ^68^Ga-VHH PD-L1; (b) ^68^Ga-VHH HER2/neu. Along Y-axis — radioactivity of 1 g tissue, the portion in percentage of the administered radioactivity. The results are represented as m±σ

The biodistribution of ^68^Ga-VHH PD-L1 or ^68^Ga-VHH HER2/neu was studied at 0.5, 1.5, and 4 h after administering into mice blood. The radionuclide ^68^Ga was found to excrete rapidly from the mice blood flow through the urinary system (Figure 3). 1.5 h later the blood radioactivity was on average no more than 2.5%. (From here on, radioactivity will be presented as a percentage of the administered dose per 1 g of tissue weight.) In addition, the significant radioactivity accumulation was found in the kidneys, it being maximum 30 min after administering ^68^Ga-VHH PD-L1 and 1.5 h after administering ^68^Ga-VHH HER2/neu. It is likely to be related to the more intensive excretion of ^68^Ga-VHH PD-L1 with urine.

**Figure 4.**
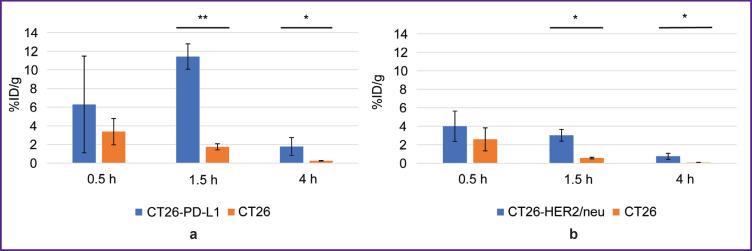
Radioactivity of the specific and non-specific tumors after administering radioimmunoconjugates: (a) ^68^Ga-VHH PD-L1; (b) ^68^Ga-VHH HER2/neu. Along Y-axis — radioactivity of 1 g tissue, the portion in percentage of the administered radioactivity. The results are represented as m±σ; * p<0.05, ** p<0.001

**Figure 5.**
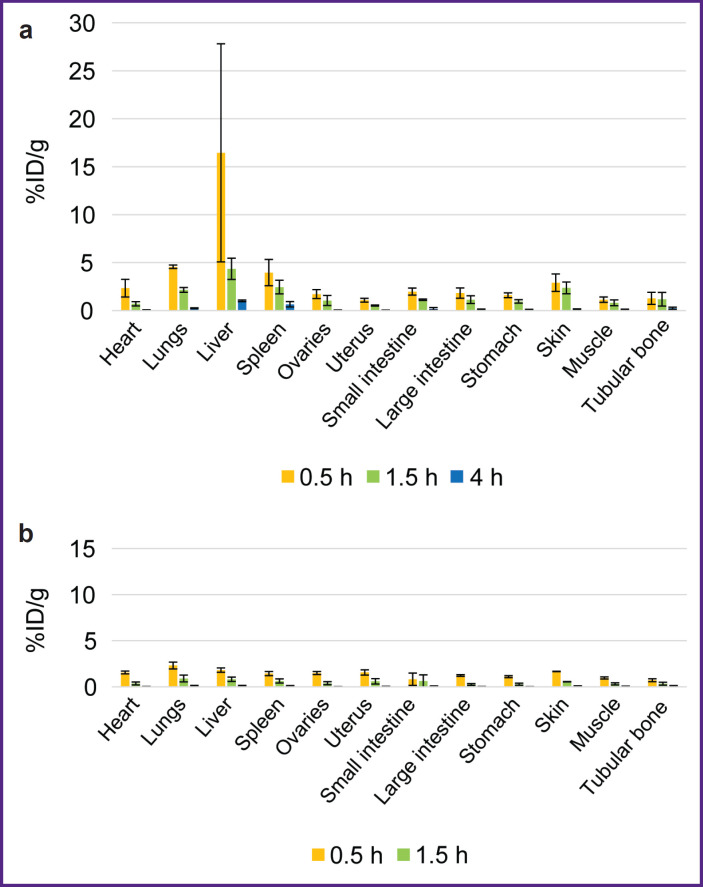
Biodistribution dynamics of radioimmuno-conjugates in the mice with tumors: (a) ^68^Ga-VHH PD-L1; (b) ^68^Ga-VHH HER2/neu. Along Y-axis — radioactivity of 1 g tissue, the portion in percentage of the administered radioactivity. The results are represented as m±σ

**Figure 6.**
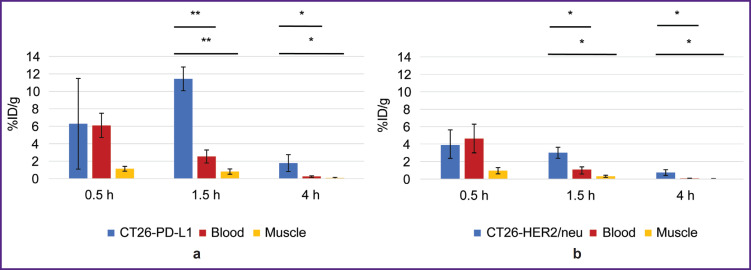
Change in the radioactivity of the specific tumor, blood, and muscular tissue after administering radioimmunoconjugates: (a) ^68^Ga-VHH PD-L1; (b) ^68^Ga-VHH HER2/neu. Along Y-axis — radioactivity of 1 g tissue, the portion in percentage of the administered radioactivity. The results are represented as m±σ; * p<0.05, ** p<0.001

1.5 and 4 h after administering ^68^Ga-VHH PD-L1 or ^68^Ga-VHH HER2/neu the specific tumor radioactivity was higher compared to that of the control (Figure 4). It showed the presence of selective RIC binding to target biomarkers PD-L1 and HER2/neu expressed by the specific tumor cells. The common pattern after administering these RICs was a rapid radioactivity decrease of the control tumor; therefore, the highest increase in the specific tumor radioactivity compared to that of the control was achieved in 1.5 h. The maximum radioactivity in the specific tumor ^68^Ga-VHH PD-L1 was on average 11.4% after ^68^Ga-VHH PD-L1 administration and 4.0% after ^68^Ga-VHH HER2/neu administration (see Figure 4). Due to the selective binding of ^68^Ga-VHH PD-L1 and ^68^Ga-VHH HER2/neu, the radioactivity in the specific tumor was higher than that in other organs and tissues, except for the kidneys (Figure 5). The difference was statistically significant for blood and muscular tissue in 1.5 and 4 h (Figure 6), in 1.5 h it being the highest. It should provide the imaging contrast in tumor diagnostics using positron-emission tomography. So, in 1.5 h after ^68^Ga-VHH PD-L1 administration, the specific tumor radioactivity 4.5 times exceeded the radioactivity in blood and in the muscular tissue — by 14.1 times. For ^68^Ga-VHH HER2/neu the excess was 3.1 and 9.4 times, respectively.

**Figure 7.**
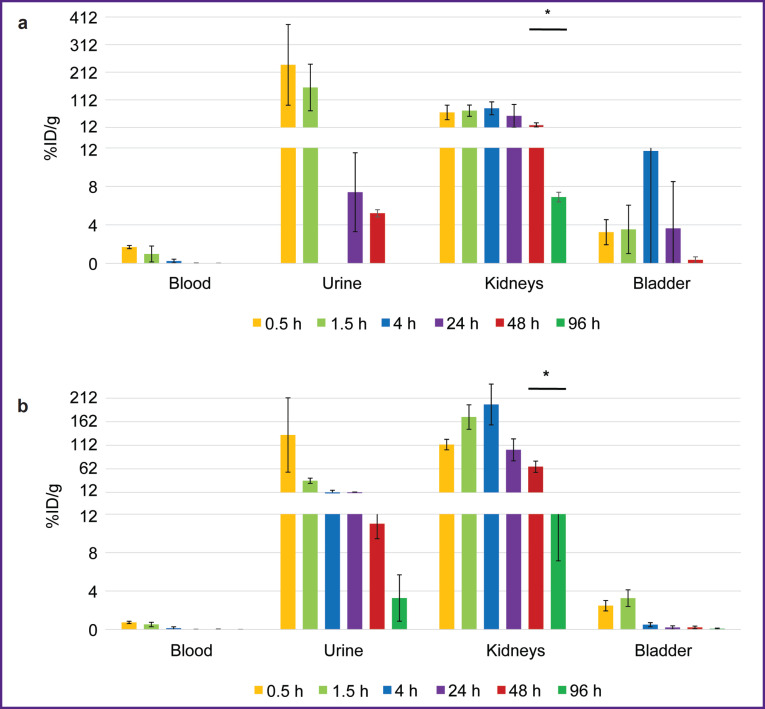
Change in the radioactivity of urine, blood, the kidneys, and the urinary bladder after administering radioimmunoconjugates: (a) ^177^Lu-VHH PD-L1; (b) ^177^Lu-VHH HER2/neu. Along Y-axis — radioactivity of 1 g tissue, the portion in percentage of the administered radioactivity. The results are represented as m±σ; * p<0.05

**Figure 8.**
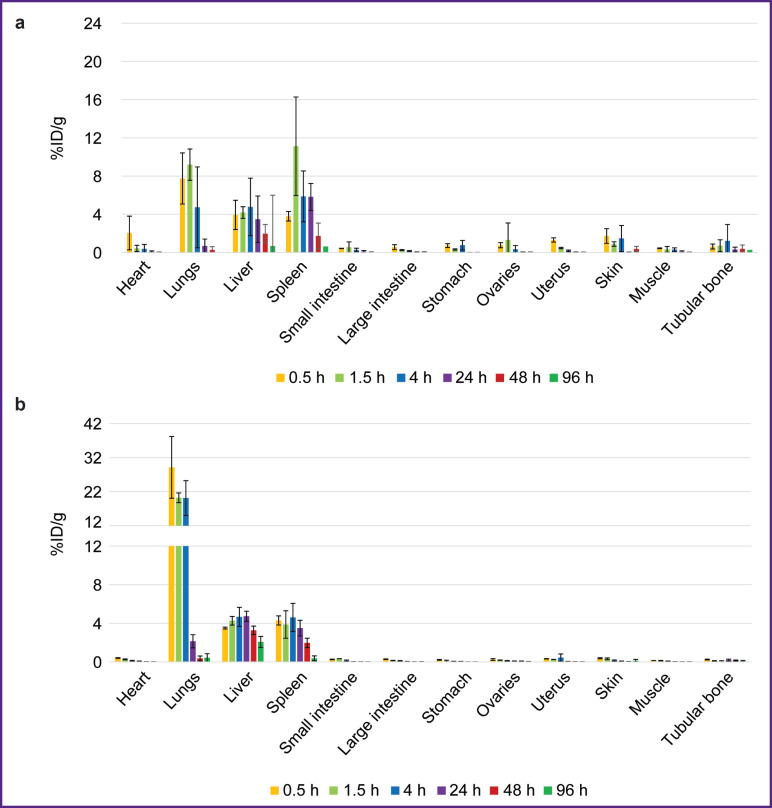
Biodistribution dynamics of radioimmunoconjugates in the mice: (a) ^177^Lu-VHH PD-L1; (b) ^177^Lu-VHH HER2/neu. Along Y-axis — radioactivity of 1 g tissue, the portion in percentage of the administered radioactivity. The results are represented as m±σ

**Figure 9.**
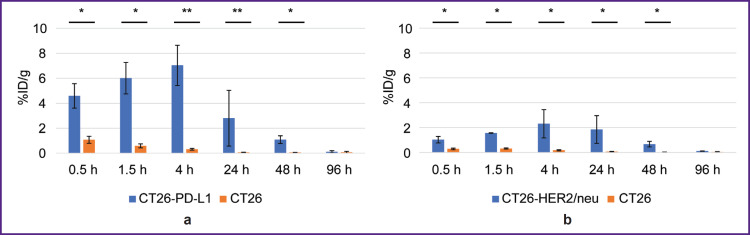
Radioactivity dynamics of the specific CT26-PD-L1 or CT26-HER2/neu tumor and the control CT26 tumor compared after radioimmunoconjugates administered: (a) ^177^Lu-VHH PD-L1; (b) ^177^Lu-VHH HER2/neu. Along Y-axis — radioactivity of 1 g tissue, the portion in percentage of the administered radioactivity. The results are represented as m±σ; * p<0.05, ** p<0.001

The biodistribution of RICs containing ^177^Lu in mice was studied within 96 h after administration, and that was due to the isotope half-life. After administering ^177^Lu-VHH PD-L1 or ^177^Lu-VHH HER2/neu, there also occurred the rapid decrease in blood radioactivity: in 4 h its value was no more than 0.25% (see Figure 6). It resulted from the fact that after administering these RICs, the radionuclides were also intensively excreted with urine, and the most part of them remained in the kidneys, where the radioactivity accumulation was maximum compared with other organs and tissues (Figures 7 and 8). The specific tumor radioactivity was comparable with (for ^177^Lu-VHH HER2/neu) or lower (^177^Lu-VHH HER2/neu) than the radioactivity of the lungs, the liver, and the spleen, although it was higher than in other organs except the kidneys (Figures 8 and 9).

Within 48 h after administering ^177^Lu-VHH PD-L1 or ^177^Lu-VHH HER2/neu, the specific tumor radioactivity exceeded the control tumor radioactivity (see Figure 9). The findings demonstrated ^177^Lu-VHH PD-L1 and ^177^Lu-VHH HER2/neu to specifically *in vivo* bind to the tumor expressing a target biomarker. The radioactivity accumulation in the specific tumor achieved its maximum in 4 h: on average 6.6% for ^177^Lu-VHH PD-L1 and 2.3% for ^177^Lu-VHH HER2/neu. Then the specific tumor radioactivity was slowly decreasing, it could provide a long-term therapeutic effect.

**Figure 10.**
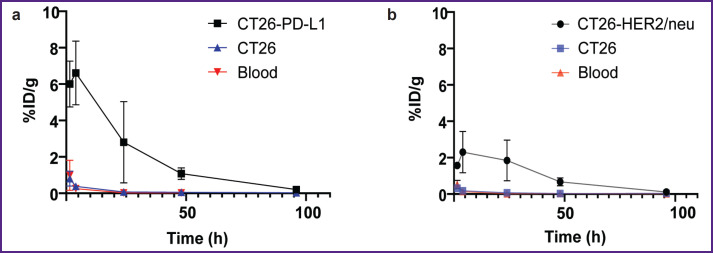
Graphs of the radioactivity dependence of the specific and control tumors and blood on the time after administering radioimmunoconjugates: (a) ^177^Lu-VHH PD-L1; (b) ^177^Lu-VHH HER2/neu

Figure 10 demonstrates the ratio of the areas under curve for the radioactivity graphs of the specific and control tumors, as well as the blood within 96 h after ^177^Lu-VHH PD-L1 and ^177^Lu-VHH HER2/neu administration. The ratios of these areas for the specific and control tumors were 19.8 after ^177^Lu-VHH PD-L1 administered and 15.6 after administering ^177^Lu-VHH HER2/neu (see the Table). Such high values confirmed the selectivity of RIC binding to the specific tumor. The ratios of such areas for the specific tumor and blood were 38.9 and 25.5, respectively. It means RIC application for therapy should result in the body being exposed to no significant radiation.

**Table:** Areas under curve (AUC, %ID/g-h) of the graphs of radioactivity dependence on the time after administering radioimmunoconjugates (m±σ), and the ratios of the specific and control tumor AUC, as well as the specific tumor and blood

^177^Lu-VHH PD-L1		^177^Lu-VHH HER2/neu	
AUC_0–96_ CT26-PD-L1	187.3±12.4	AUC_0–96_ HER2/neu	95.3±9.7
AUC_0–96_ CT26	9.4±0.8	AUC_0–96_ CT26	6.1±0.5
AUC_0–96_ blood	4.7±0.4	AUC_0–96_ blood	3.7±0.3
AUC_0–96_ CT26-PD-L1/ AUC_0–96_ CT26	19.8	AUC_0–96_ CT26-HER2/neu/ AUC_0–96_ CT26	15.6
AUC_0–96_ CT26-PD-L1/ AUC_0–96_ blood	38.9	AUC_0–96_ CT26-HER2/neu/ AUC_0–96_ blood	25.5

**Figure 11.**
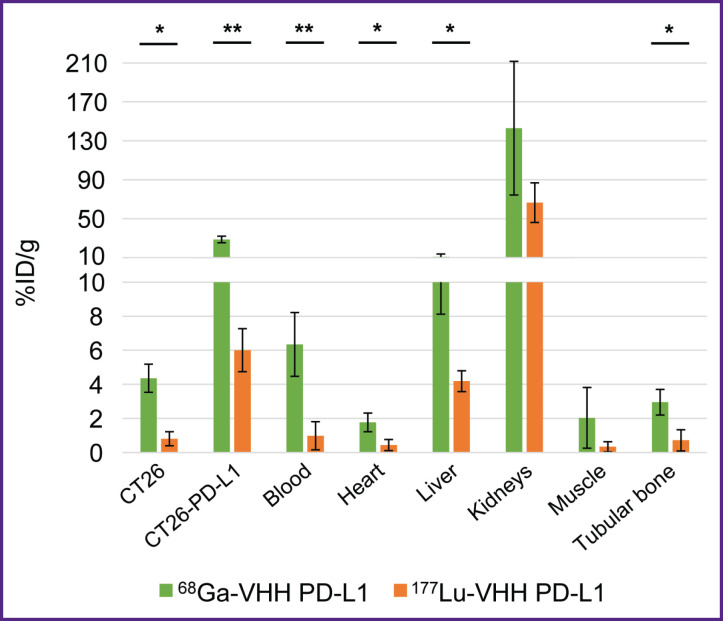
Biodistribution of ^68^Ga-VHH PD-L1 and ^177^Lu-VHH PD-L1 1.5 h after administration Along Y-axis — radioactivity of 1 g tissue, the portion in percentage of the administered radioactivity. The results are represented as m±σ; * p<0.05, ** p<0.001

The present study investigated the dependence of RIC biodistribution of both: the nanobodies constituting them and also the radionuclides, since in literature we found no data comparing the biodistribution of RIC containing the same nanobodies with these radionuclides. We compared the radioactivity of mice organs and tissues in 1.5 h after ^68^Ga-VHH PD-L1 and ^177^Lu-VHH PD-L1 administration. To make the comparison, there was taken into consideration the rapid decay of ^68^Ga through the radioactivity normalization over the administration time. The accumulation of ^68^Ga-labeled nanobodies in the specific and non-specific tumors, blood, the heart, and the liver was shown to multiply exceed the accumulation of ^177^Lu-labeled antibodies (Figure 11). It is of importance to note that these preparations were based on one nanobody and had one chelating agent DOTA that reduces the interpretation variants of the findings.

## Discussion

The main objective of the study was to demonstrate the applicability of RIC pairs of nanobodies with radioisotopes for theranostics of tumors expressing PD-L1 and HER2/neu biomarkers. The nanobodies were conjugated with ^68^Ga, used for diagnostic purposes, and with ^177^Lu, used for tumor therapy. The findings showed the presence of specific binding of all RICs to the tumors expressing PD-L1 or HER2/neu, respectively. After administering these RICs into the blood flow, there occurred the intensive elimination of radionuclides ^68^Ga and ^177^Lu through the urinary system. It was due to the fact that the molecular weight of protein molecules in RICs is lower than the threshold weight equaled 65 kDa. Due to this, these molecules can pass through the renal barrier and be excreted with urine [28, 29]. As a result, there was the sharp drop of radioactivity in organs and tissues. The fragments of the antibodies, including VHH, are known to be removed from blood more rapidly than the whole monoclonal antibodies, the half-life of which is several days due to their large molecular weight (150 kDa) and due to this — the impossibility to pass through the renal filter from blood into the primary urine [30–32]. One more advantage of RICs based on nanobodies and other low-molecular-weight vector molecules is no Fc fragment. This fragment of the whole monoclonal antibodies binds in the body to the neonatal receptor of Fc fragment (FcRn) expressed by the body cells, including endothelial cells of blood vessels, it preventing the monoclonal antibodies in lysosomes after endocytosis from the degradation, and resulting in their recirculation back to the cell surface and, therefore, in blood delay [33, 34]. In addition, such RICs do not have the effector functions caused by this fragment, which are necessary for the functioning of the immune system, but in some cases may be undesirable for radiopharmaceuticals [35, 36].

The lower molecular weight of nanobodies and other vector molecules underlies one more feature of the pharmacokinetics compared to whole antibodies. Apart from the intensive excretion from the body with urine, VHH and other fragments more rapidly and uniformly penetrate the specific tumor tissue [37]. Due to the combination of these properties, there is the rapid and significant radioactivity excess of the tumor expressing target biomarkers over the radioactivity of blood and muscular tissue. It enables to use RICs with short-lived isotopes, such as ^68^Ga, for contrast tumor imaging. The rapid elimination of radionuclides from the blood flow also leads to the radiation load decrease in tumor therapy. The previously studied radioconjugates based on small scaffold proteins and antibody fragments were eliminated from the blood faster, and more accumulated in the kidneys compared to whole antibodies [18–22].

One of the problems in using RICs based on nanobodies is the relatively high clearance rate of radionuclides from a specific tumor: 96 h after the administration of such RICs containing ^177^Lu the tumor radioactivity was very low (see Figure 9). It resulted from the rapid RIC elimination from the blood, and due to this only a small amount of labeled nanobodies reached their target. To achieve a high target radiation load, RIC can be administered multiple times [38]. Another problem is the radiation accumulated in the kidneys. The elimination of RICs based on nanobodies through the urinary system results in the significant exposure of these organs, it is likely to be due to the fact that metal-chelate complexes retain in lysosomes of the epithelial cells in renal tubules when reabsorbing from the primary urine [28]. It is primarily urgent for RICs containing radionuclides used for therapy, since their radiation can damage the body tissues rather than the radiation of radionuclides applied for diagnostics. Currently, in the clinic, arginine and lysine are administered as accompanying substances along with some radiopharmaceuticals to reduce radioactivity in the kidneys. These substances competing against radiopharmaceuticals for binding sites, used in reabsorption, reduce the radioactivity accumulation in the kidneys. Other approaches to solve the problem are also being under study at present, including preliminary targeting, attaching albumin-binding domain, using cleavable linkers [39–41].

The represented findings have demonstrated the applicability of using RIC pairs of ^68^Ga-VHH PD-L1 and ^177^Lu-VHH PD-L1, ^68^Ga-VHH HER2/neu and ^177^Lu-VHH HER2/neu for the theranostics of the tumors expressing PD-L1 and HER2/neu. In each pair, RICs differed only in radionuclides, but not in vector molecules and chelating agents. Using such pairs will make easier the RIC production standardization, simplify the treatment process logistics, and reduce its cost. The previous studies [23, 42–45] of conjugates of the radionuclides with immune vector molecules specific to PD-L1 or HER2/neu did not provide such approach.

In contrast to the widely used in theranostics radioconjuagates based on peptides and other low-molecular ligands, nanobodies can be selective for any epitopes. Therefore, one might expect to obtain new nanobody-based theranostic RIC pairs specific to other biomarkers. Moreover, RICs can contain other isotopes intended for diagnostics or therapy. Radionuclides vary in radiation, a half-life period, and toxicity that enables to choose an appropriate isotope in developing a radiopharmaceutical designed for solving a certain task. Thus, a need arises to study and compare a great number of RICs different in vector molecules and/or radionuclides. It actualizes to use in preclinical trials standardized, defined, reproducible, and advisably inexpensive experimental models. Such models are genetically engineered cells based on mice tumor cells, and carrying human tumor biomarkers, which can be inoculated to mice with the minimally modified or intact immune system [27, 46, 47]. As noted, in the present study, to form a tumor, the mice were inoculated with the humanized cells CT26-PD-L1 or CT26-HER2/neu based on murine CT26 cells, and expressing human PD-L1 and HER2/neu, respectively.

An interesting and unexpected observation was made: the biodistribution of RICs under study in organs and tissues depended on both — the nanobodies they were composed of and also radionuclides. The accumulation of ^68^Ga-labeled nanobodies in some organs and tissues, including tumors, was shown to multiply exceed the accumulation of the same nanobodies labeled with ^177^Lu. The biodistribution of the radiopharmaceuticals containing radioactive isotopes of metals and halogens is known to be different, since halogens less retain in the kidneys [28]. The present study revealed the significant difference in the biodistribution of RICs containing the isotopes of different metals.

## Conclusion

The study demonstrated the functional suitability of RIC pairs of nanobodies with radioisotopes ^68^Ga and ^177^Lu for the diagnostics and therapy of the tumors expressing PD-L1 and HER2/neu biomarkers. Each RIC pair with the same specificity was synthesized using one and the same vector molecule and one and the same chelating agent. It enables to improve therapy reliability and reduce the treatment cost. The development of such theranostic pairs of radiopharmaceuticals based on the fragments of monoclonal antibodies can open great capabilities for the effective personalized cancer treatment.
